# The – Not So – Solid 5.5 cm Threshold for Abdominal Aortic Aneurysm Repair: Facts, Misinterpretations, and Future Directions

**DOI:** 10.3389/fsurg.2016.00001

**Published:** 2016-01-25

**Authors:** Nikolaos Kontopodis, Dimitrios Pantidis, Athansios Dedes, Nikolaos Daskalakis, Christos V. Ioannou

**Affiliations:** ^1^Vascular Surgery Unit, University Hospital of Heraklion, University of Crete Medical School, Crete, Greece; ^2^Vascular Surgery Department, Red Cross Hospital, Athens, Greece

**Keywords:** abdominal aortic aneurysm, indication for aneurysm repair, computational analysis, future prospective for AAA repair, aneurysm geometry, aneurysm wall stress, intraluminal aortic thrombus

## Abstract

Abdominal aortic aneurysms (AAAs) represent a focal dilation of the aorta exceeding 1.5 times its normal diameter. It is reported that 4–8% of men and 0.5–1% of women above 50 years of age bear an AAA. Rupture represents the most disastrous complication of aneurysmal disease that is accompanied by an overall mortality of 80%. Autopsy data have shown that nearly 13% of AAAs with a maximum diameter ≤5 cm were ruptured and 60% of the AAAs >5 cm in diameter never ruptured. It is therefore obvious that the “maximum diameter criterion,” as a single parameter that fits all patients, is obsolete. Investigators have begun a search for more reliable rupture risk markers for AAA expansion, such as the level and change of peak wall stress or AAA geometry. Furthermore, it is becoming more and more evident that intraluminal thrombus (ILT), which is present in 75% of all AAAs, affects AAA features and promotes their expansion. Though these hemodynamic properties of AAAs are significant and seem to better describe rupture risk, they are in need of specialized equipment and software and demand time for processing making them difficult in use and unattractive to clinicians in everyday practice. In the search for the addition of other risk factors or user-friendly tools, which may predict AAA expansion and rupture, the use of the asymmetrical ILT deposition index seems appealing since it has been reported to identify AAAs that may have an increased or decreased growth rate.

## Size: A Critical Determinant of Rupture Risk

Abdominal aortic aneurysm (AAA) is a common encountered pathology in the aging population of the developed countries (1, 2). Early enough, it was recognized that the main risk factor to predict the catastrophic event of aneurysm rupture was its size, mainly with respect to its maximum diameter. This realization was based on both physical principles, namely the law of Laplace, and clinical practice/experience of surgeons dealing with such patients. The law of Laplace is usually pointed as the theoretical basis for using the “maximum diameter criterion” to predict AAA rupture risk (3). This “law” was developed about 200 years ago and provides explanation of the mechanisms of a wide range of physiologic phenomena. Regarding the circulatory system, it states that the stress exerted on the AAA wall is proportional to its diameter, or in other words, the larger the aneurysm, the higher the risk of rupture (4).

As early as from the 60s, the medical literature has recorded that small non-operated aneurysms (<6 cm) presented a significantly better survival rate than their larger (again non-operated) counterparts (>6 cm). Moreover, since the definition of “small” employed AAAs <6 cm it is not surprising that even in this category, surgery offered a significant survival advantage compared to surveillance (5). Thereafter, several reports have confirmed the fact that increased size may serve as the most significant risk factor to foretell a high risk of rupture (6, 7).

## Defining Cut-Off Values to Determine AAA Therapeutic Management

### Landmark Studies

It took several years of research, thousands of patients, and two randomized control trials (RCTs) to determine the exact cut-off value where the risk of rupture exceeded operative risks from open surgical repair and therefore elective surgery would be justified. The United Kingdom Small Aneurysm Trial (UKSAT) performed in the UK, included 1090 patients the first recruited in 1991 (8). The aneurysm detection and management (ADAM) trial included 1136 patients the first recruited in 1992 (9). These trials presented their results in 1998 and 2002, respectively, and both came down to the conclusion that survival is not improved by open elective repair of AAAs smaller than 5.5 cm. At this diameter, the risk of surgical repair outweighs the risk of rupture during surveillance. Therefore, according to these results, surveillance of small AAAs would be safe and advisable compared to early surgery.

### Technological Progress – The Advent of Endovascular Repair

Rapid progress in the field of medical technology along with the intuitive minds and daring of some very important pioneers in the endovascular field, brought treatment modalities in the next level with the introduction of endovascular aneurysm repair (EVAR). Taking into account that open surgery did not appear to be beneficial until the diameter of the aneurysm reached >5.5 cm along with data from clinical trials, which confirmed a lower risk of operative mortality after EVAR and also suggested that EVAR outcome is directly related to aneurysm size being better for smaller aneurysms, a clinical trial testing the hypothesis that EVAR is beneficial in patients with small AAA was warranted. Two RCTs, such as the positive impact of endovascular options for treating aneurysms early investigators (PIVOTAL) (728 participants) and the comparison of surveillance vs. aortic endografting for small aneurysm repair studies (CAESAR) (360 participants), tested the hypothesis that early EVAR may be beneficial compared to surveillance (10, 11). Their results were published at 2010 in JVS and 2011 in EJVES and both studies came down to the conclusion that mortality and rupture rates in small AAAs are low and no clear advantage could be demonstrated between early or delayed EVAR strategy.

## Pitfalls and Flaws in the Interpretation of RCTs

According to the abovementioned data, someone would conclude that there is enough evidence to support a watchful waiting for small AAAs and a prompt repair for larger ones using the solid criterion of 5.5 cm as the cut-off value. Nevertheless, a fact that commonly escapes attention and is definitively worth noting, is that landmark studies comparing open surgery vs. surveillance took place during the 90s and therefore they were hampered by the absence of thin-slice computed tomography (CT), digital imaging, and the three-dimensional (3D) reconstruction of AAA surface, which are currently widely available. Subsequently, the UKSAT was based on ultrasonographic measurements of the aneurysm anteroposterior maximum diameter, while the ADAM used axial CT measurements (8, 9). On the other hand, the more recent studies examining EVAR for small AAAs have used orthogonal maximum diameter measurements meaning perpendicular to the centerline of flow as currently recommended by the SVS guidelines (10–12). Naturally, these different modes used to record aneurysm size are not necessarily equivalent and may pose inaccuracies in therapeutic management of AAA patients. Sprouse et al. compared between US, axial CT and orthogonal CT measurements. These authors not only recorded significantly larger diameter when measurements were made in an axial plane compared to orthogonal CT and US measurements but also indicated that limits of agreement between measurements were poor and exceeded clinical acceptability (5 mm) and therefore could result in therapeutic inaccuracies (13). Others have compared between US and orthogonal CT measurements and found a consistently larger recording with the latter modality (mean difference 0.21 cm), while again the limits of agreement were −0.55 to 0.96 cm, exceeding clinical acceptability. Notably, 70% of those patients with a US recording between 5 and 5.5 cm, which would suggest conservative management, had CT scans revealing diameters >5.5 cm which on the contrary would set the indication for surgical correction (14). Moreover, a recent study, which compared between ultrasound and CT maximum diameter measurements in both axial and orthogonal planes, indicated that the mean of each series of readings on CT was significantly larger than the mean US measurement, and that CT measurements also differed significantly from each other. The axial CT diameter resulted in the larger recordings, which exceeded orthogonal measurements by a mean of 2.4 ± 5 mm, while the US diameter was smaller than CT axial by 9.6 ± 8.0 mm and CT orthogonal diameter by 7.3 ± 7.0 mm (15). In line with these results, what would have been a 4.0–5.5 cm (target group under investigation) AAA according to the UKSAT measurements would be recorded as a 4.9–6.4 cm according to reporting standards of ADAM and actually would be a 4.7–6.2 cm aneurysm according to contemporary methods. Of course, these discrepancies emerge by the extrapolation of the differences between different modes of diameter measurements to the cut-off values and measurement methods that the UKSAT and ADAM trials used and therefore provide an estimation of possible inaccuracies. Of course, we do not suggest that early studies lose their significance but believe that their results may not be suitable to be simply applied in current clinical practice and should be interpreted with caution. In other words, the technological advancements in medical imaging have rendered previously described measuring methods out of date, in the same time that evidence-based recommendations are still based on studies using less sophisticated techniques.

Another pitfall that may be reported is the fact that the two studies comparing EVAR vs. surveillance for the small AAAs refer to different study populations. In fact, the PIVOTAL study included AAAs in the range of 4.0–5.0 cm, while the CAESAR study recruited subjects with AAA measuring 4.1–5.4 cm. Therefore, a subpopulation of patients with AAAs of maximum diameter between 5.1 and 5.4 cm were included in the CAESAR, but not in the PIVOTAL study. Since this is the subgroup of patients where the greater number of ruptures throughout the surveillance period would be expected, a surprisingly low rupture rate (0.3%) in the PIVOTAL study may not be that surprising after all, while the higher rate of subsequent AAA repairs in the surveillance group in the CAESAR study (60 vs. 31%) may be expected. Overall, the abovementioned data may indicate that the results of these studies probably are not absolutely comparable to each other, and therefore their interpretation should not be generalized (10, 11).

## Risk Determinants Beyond the “*One-Size Fits All*” Maximum Diameter Criterion

There is now a significant body of evidence to suggest that besides maximum diameter other factors may play a significant role in the AAAs natural history toward enlargement and rupture (16). This may not be so surprising after all, if someone considers that diameter matched AAAs may present several differences like in geometrical indices (length, angulation, tortuosity, curvature, shape), AAA volume, intraluminal thrombus (ILT) thickness/volume/distribution, wall stress, and wall strength (17–25). It would seem naive for someone to expect that none of these factors would have anything to do with the AAAs rupture risk and that the latter could definitively be determined based on the sole criterion of maximum diameter. Therefore, several researchers have suggested that a greater amount of ILT may pose a greater risk of rupture mainly by reducing the O_2_/nutrient delivery and promoting an inflammation process to the arterial wall, while a predominantly posterior deposition of ILT has been shown to be correlated with an increased growth rate (26–29). Others postulated that increased cross-sectional asymmetry and decreased tortuosity may foretell a higher rupture risk in the same time that the mean annual growth rate may be significantly lower in men with an AAA wall calcification >50% of its circumference (30, 31). More importantly, currently, it is well understood that rupture is a localized phenomenon that occurs when and where the local stress exerted to the aneurysmal wall due to systemic pressurization exceeds its strength (3, 16). Accordingly, there are now enough data in the literature to suggest that biomechanical indices, i.e., peak wall stress and peak wall rupture index are far superior than maximum diameter alone in predicting the AAAs rupture risk potential (32). Moreover, it now seems that the time when calculation of these indices will not to be performed in specialized labs but will be widely available and accessible to clinicians, is not far away (33, 34). In this context, where a point-wise comparison of wall stress and strength will be possible and a mapping of the rupture risk along the aneurysmal surface may be performed the current gold standard, maximum diameter criterion, will probably become obsolete.

## Intraluminal Thrombus – The Asymmetrical Thrombus Deposition Index

In the search for more reliable rupture risk markers, ILT is one of the most studied AAA features, which has been shown to correlate well with AAA expansion and is present in 75% of all AAAs (35). Researchers have previously shown that ILT load is associated with a higher possibility of AAA expansion (36); furthermore, an increase of thrombus cross-sectional area may predict AAA rupture better than simple maximum diameter increase (37). Additionally, ILT thickness has demonstrated a positive correlation with the risk of rupture (38), and it has been observed that greater volumes of ILT were noted in ruptured as compared to elective AAA cases (39). An extensive list of publications on ILT effect on rupture risk currently exist but the majority focus on ILT size-related characteristics (35, 38–40). These studies’ main focus were on volume/area growth, relative sac volumes, and maximum thickness’s. Only a few reports exist regarding the effect of ILT spatial distribution on AAA rupture risk (35, 38–40).

**Figure 1:**
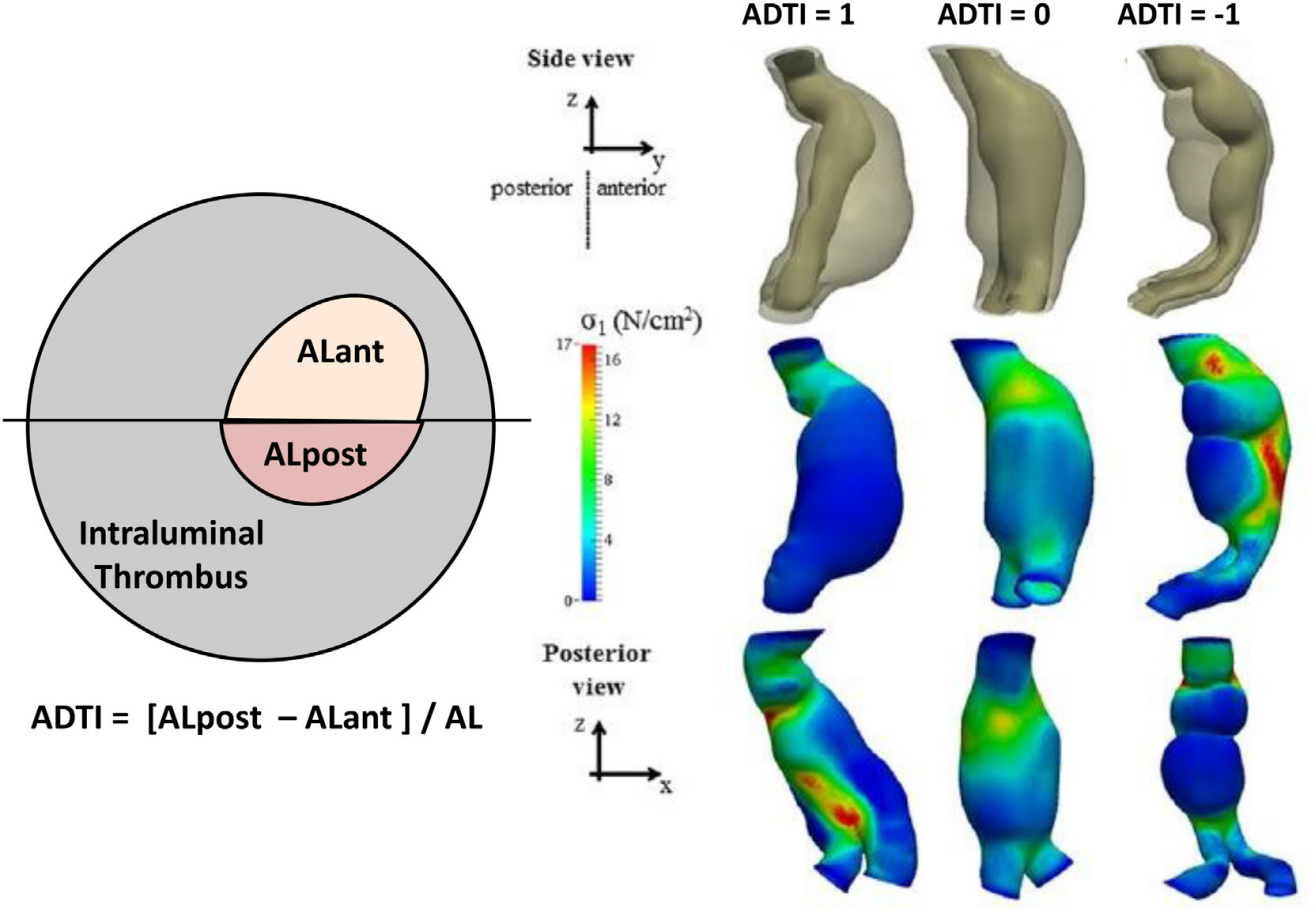
**Definition of asymmetrical thrombus deposition index (ATDI)**. In this example, ATDI is negative. The black line determines the anterior and posterior sides, and the luminal areas at the anterior (ALant) and the posterior (ALpost) sides are measured. Also depicted are three examples of different ADTI ranging from +1 to −1 along with their wall stress distribution. Figure modified from Ref. (28).

Most AAAs actually present an asymmetrical ILT deposition (39). Previous studies have suggested site-specific regional differences in wall biochemistry in the aorta (41, 42). Could the spatial distribution of ILT have an effect on AAA progression? Recently, Metaxa et al. attempted to answer this question (28). They hypothesized that ILT may have a different effect on AAA progression depending on its deposition pattern (28). Metaxa et al. examined whether asymmetrical ILT deposition is associated with growth rate (Figure 1). They found that aneurysms with negative asymmetrical thrombus deposition index (ATDI) (meaning a posteriorly located ILT) had a significantly lower growth rate compared to those with positive ATDI (an anteriorly located ILT) (28). This result could be useful in clinical practice as asymmetrical posterior ILT deposition can be easily determined qualitatively from a common CT scan without any special computerized modeling. The theoretical basis behind these findings may be the fact that thrombus has been shown to lower wall stress (43), but at the same time, it is a biologically active laminated structure that can influence proteolysis in the underlying wall (44). Although thick ILT depositions may not directly cause harm to the adjacent AAA wall regions since ILT’s proteolytic activity is predominantly located in the luminal layer (44), thick ILT depositions could cause local hypoxia to the AAA sac wall (45). The better oxygenation of the posterior wall via the vasa vasorum that originate from the lumbar arteries may protect it from the degenerative influence of thrombus, which may not be the case when ILT is located anteriorly (46, 47). On the other hand, AAAs with anterior ILT deposition tend to present a redistribution of wall stress against the posterior wall where regions of high stress are observed. Accordingly, one could hypothesize that rupture on the anterior wall mainly is due to reduced strength and rupture on the posterior wall is due to increased wall stress. Since the majority of ruptures occur on the posterior and/or posterolateral wall regions, posterior wall stress has been highlighted recently as potentially significant in rupture risk estimation (48–52). Accordingly, both the degeneration of the anterior wall and the concentration of wall stress to the posterior wall may render AAAs with anterior ILT more susceptible to rupture. Of course, such conclusions need further scientific proof to be established and gain clinical applicability. Overall, a low rupture risk marker could increase the time intervals between CT scans, reducing health costs to providers, and any additional anxiety to the patients (28). It may also potentially reduce any unnecessary interventions, cost, and perioperative morbidity and mortality that accompany surgical treatment of AAAs with a diameter >5.5 cm. Additionally, the degree of asymmetrical ILT deposition, measured by the ATDI, could be integrated in a multimodal rupture risk model to improve rupture risk stratification (28).

## Conclusion

Currently, the 5.5-cm criterion is a well-respected threshold to set the indication for AAA elective repair, which is widely used to determine therapeutic management of these patients. Nevertheless and despite the fact that currently SVS recommendations require 3D reconstruction in order to record maximum diameter in a plane perpendicular to the centerline of flow, diameters measured in this way have not previously been used in the landmark studies and therefore may not be absolutely and correctly correlated with current treatment indications. The addition of ILT status into the estimation of possible rupture risk seems applicable and needs further investigation. Moreover, rapid advancements in medical imaging and post-processing and computational analysis have given access to several parameters that may influence AAA rupture risk. Hopefully, a pinpoint comparison of wall stress and strength throughout the aneurysmal surface will soon become possible and widely available which then will make the 5.5-cm diameter criterion obsolete or outdated.

## Author Contributions

NK: performed literature search and wrote the initial draft. DP: performed the literature search. AD: performed the literature search. ND: assisted in writing the initial draft. CI: performed critical revision of the paper and had overall responsibility.

## Conflict of Interest Statement

The authors declare that the research was conducted in the absence of any commercial or financial relationships that could be construed as a potential conflict of interest.
